# The Development of Whole Sporozoite Vaccines for *Plasmodium falciparum* Malaria

**DOI:** 10.3389/fimmu.2018.02748

**Published:** 2018-12-11

**Authors:** Leslie S. Itsara, Yaxian Zhou, Julie Do, Alexis M. Grieser, Ashley M. Vaughan, Anil K. Ghosh

**Affiliations:** ^1^MalarVx, Inc., Seattle, WA, United States; ^2^Seattle Children's Research Institute, Seattle, WA, United States

**Keywords:** *Plasmodium falciparum*, malaria, whole sporozoite vaccines, genetically attenuated parasite, radiation attenuated sporozoite, *in vitro* culturing

## Abstract

Each year malaria kills hundreds of thousands of people and infects hundreds of millions of people despite current control measures. An effective malaria vaccine will likely be necessary to aid in malaria eradication. Vaccination using whole sporozoites provides an increased repertoire of immunogens compared to subunit vaccines across at least two life cycle stages of the parasite, the extracellular sporozoite, and intracellular liver stage. Three potential whole sporozoite vaccine approaches are under development and include genetically attenuated parasites, radiation attenuated sporozoites, and wild-type sporozoites administered in combination with chemoprophylaxis. Pre-clinical and clinical studies have demonstrated whole sporozoite vaccine immunogenicity, including humoral and cellular immunity and a range of vaccine efficacy that depends on the pre-exposure of vaccinated individuals. While whole sporozoite vaccines can provide protection against malaria in some cases, more recent studies in malaria-endemic regions demonstrate the need for improvements. Moreover, challenges remain in manufacturing large quantities of sporozoites for vaccine commercialization. A promising solution to the whole sporozoite manufacturing challenge is *in vitro* culturing methodology, which has been described for several *Plasmodium* species, including the major disease-causing human malaria parasite, *Plasmodium falciparum*. Here, we review whole sporozoite vaccine immunogenicity and *in vitro* culturing platforms for sporozoite production.

## Introduction

According to the 2016 World Malaria Report by the World Health Organization (WHO), nearly half of the world's population live in areas at risk of malaria transmission (1). The people most impacted by malaria are children under the age of five and pregnant women living in sub-Saharan Africa. In 2016, *Plasmodium falciparum* (Pf) caused the majority of the 445 thousand deaths and more than 200 million clinical cases, despite recent progress in malaria control efforts, which include vector control with insecticidal bed-nets and insecticide spray and chemoprevention with anti-malarial drugs (1). While these efforts have reduced morbidity and mortality caused by the disease, their impact has declined since the number of deaths and clinical cases have remained constant in the recent years highlighting the need for an effective vaccine. Vaccines represent the most effective form of control for certain infectious disease as demonstrated by the eradication of smallpox and near eradication of polio. However, vaccine development against *Plasmodium* has proved more challenging than vaccine development against simpler bacteria or viruses. *Plasmodia* are complex eukaryotic parasites with large genomes (5,300 genes) and transform through multiple life cycle stages with stage-specific gene (antigen) expression (2). Thus, malaria vaccine development has been more challenging. For example, RTS, S/AS-01 (RTS, S) the most developed malaria vaccine candidate, which elicits antibodies against the major sporozoite (SPZ) surface protein, circumsporozoite protein (CSP), only provides partial efficacy (3, 4).

## Whole Sporozoite Malaria Vaccines in Development

**Figure 1:**
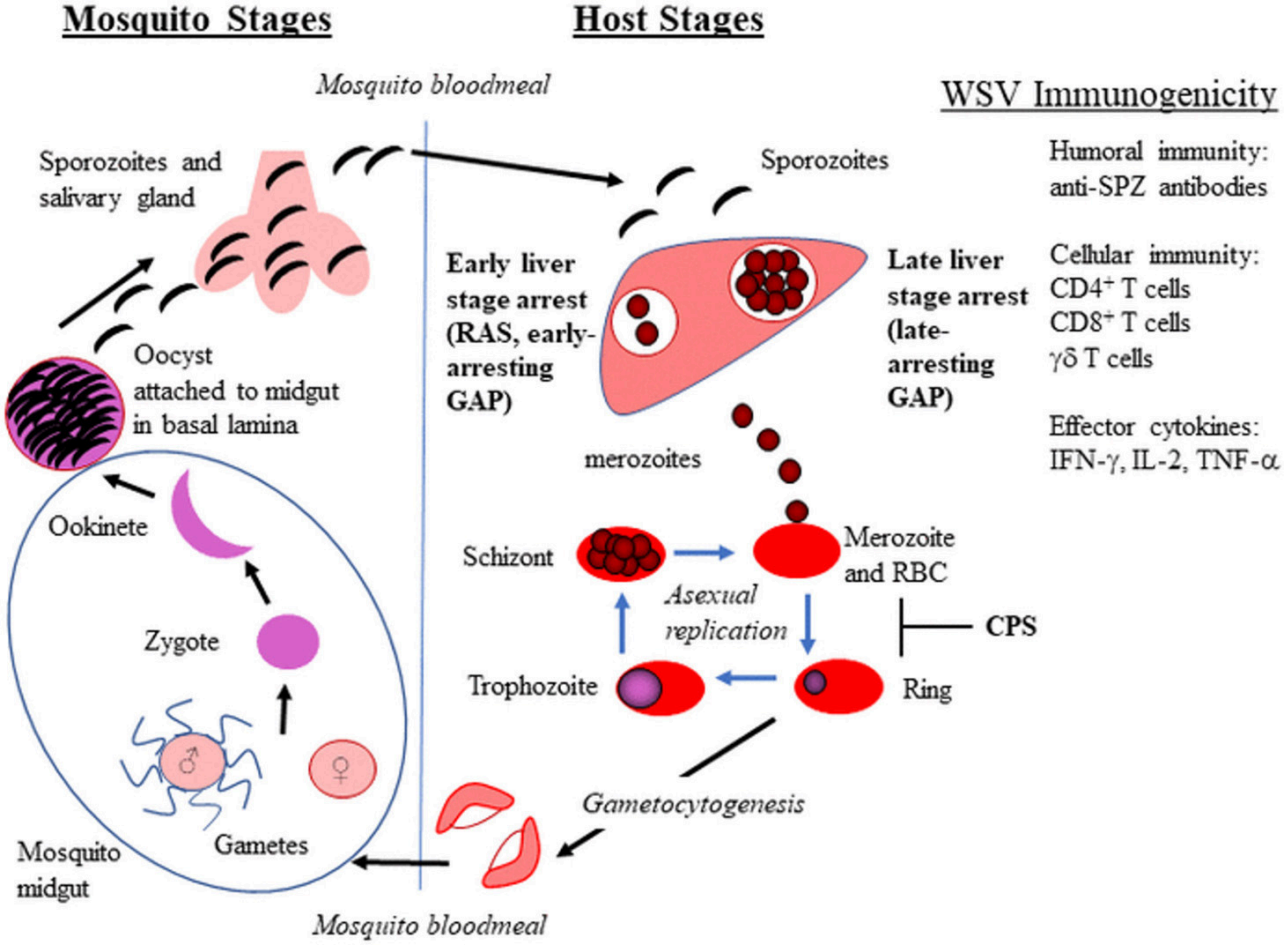
*Plasmodium* life cycle in mosquitoes and vertebrate hosts. The whole sporozite vaccine (WSV) strategy for radiation attenuated sporozoites (RAS), genetically attenuated parasites (GAP), and chemoprophylaxis with WT sporozoites (CPS) is shown in bold. The WSV immunogenicity provided by WSV administration is also listed.

Whole sporozoite vaccines (WSV) have demonstrated a range of vaccine efficacy that depends on prior exposure to malaria and ranges from ~35 to 100% efficacy, in which malaria naïve volunteers are better protected. WSV in development include radiation attenuated sporozoites (RAS), genetically attenuated parasites (GAP), and WT sporozoites administered under drug cover (known as CPS–chemical prophylaxis with SPZ, ITV–infection treatment vaccination, or CVac–using Sanaria's injectable SPZ; hereafter referred to as CPS) (Figure 1). These live SPZ infect hepatocytes but do not lead to an established blood stage infection either by arresting in liver stage development (RAS and GAP) or by being eliminated during the initial stages of red blood cell (RBC) infection (CPS). WSV elicit immunity during two stages of the parasite life cycle, extracellular SPZ and intracellular liver stage for GAP and RAS and up to the initial stages of RBC infection for CPS. Thus, WSV can elicit immunity without causing clinical malaria symptoms. RAS delivered by bites of irradiated mosquitoes were first tested in humans in 1973 and protected humans from *Pf* malaria challenge (5). Since then, Hoffman and colleagues have made significant progress to produce vialed SPZ from aseptic mosquitoes for clinical testing of RAS (PfSPZ Vaccine) and CVac [Reviewed in (6)]. A recent PfSPZ study involving malaria-experienced individuals in Mali, in which 66% of the Malian vaccine group developed malaria infection, demonstrated reduced efficacy compared to trials involving malaria-naïve individuals (7). Thus, the PfSPZ vaccine failed to provide adequate protection in pre-exposed individuals. This study highlights the need for an improved dosing strategy, immunogenicity enhancement, and/or alternative vaccine approach for populations in malarious regions.

Alternative approaches include other WSV types described here and immunogenicity enhancement of WSV and subunit vaccines described in the following section (Whole Sporozoite Vaccine Immunogenicity). Current GAP in clinical development include early-arresting liver stage parasites that arrest at a similar developmental stage as RAS (< 24 h) (8, 9). More recent *P. yoelii* pre-clinical GAP studies have engineered late-arresting liver stage GAP (2–3 days) (10–12). Whole parasites that arrest later in liver stage development (late-arresting GAP) or progress to the blood stage (CPS), provide a larger biomass and broader repertoire of immunogens, permitting lower SPZ doses for protection. For example, clinical CPS studies showed volunteers required 10- to 100-fold fewer parasites than RAS to achieve complete protection against malaria challenge, and pre-clinical late-arresting GAP studies showed that at least 10-fold fewer parasites could provide sterilizing protection and also protect against blood stage challenge (12, 13). Broad protection of late-arresting GAP was demonstrated by immunization with late-arresting *P. yoelii* GAP, which protected against cross-species challenge using *P. berghei* SPZ (11); however, heterologous challenge after CPS vaccination resulted in only modest protection after challenge (14). *Pf* GAP3KO safety has been demonstrated in a human clinical trial in which parasites arrested during liver stage development as all ten volunteers remained blood stage negative (8). However, in a previous safety trial using the first developed GAP, GAP2KO (*p36*^−^ and *p52*^−^) one in six volunteers showed patent blood stage infection (15). GAP2KO arrest later in liver stage development compared to GAP3KO (16), thus highlighting the challenge in developing a late-arresting GAP that is also completely liver-stage attenuated. Nevertheless, several groups are working on the development of late-arresting GAP due to their potential benefits.

## Whole Sporozoite Vaccine Immunogenicity

Pre-clinical and clinical studies using GAP, RAS, and CPS have demonstrated safety of vaccination with WSV, immunogenicity, and vaccine efficacy [(17); Reviewed in (6, 8, 18). WSV immunogenicity data supports CD8^+^ T cells as playing a critical role in protection. CD4^+^ T cells, γδ T cells, and antibodies are also elicited after immunization, but their roles in the protective immune response are less clear. Complete reviews focused on WSV immunogenicity and potential clinical correlates of protection are discussed elsewhere (19, 20).

### Cellular Immunity: CD8^+^, and γδ T Cells

Numerous studies support roles for cellular immunity in WSV protection, including CD8^+^, γδ, and CD4^+^ T cells. The strongest immunological data supports roles for cytokine-producing CD8^+^ T cells, liver resident CD8^+^ T cells, and γδ T cells as possible WSV immune correlates during liver stage infection. For example, in rodents, depletion of interferon-gamma (IFN-γ) or CD8^+^ T cells blocked RAS mediated sterile immunity (21). Subsequent studies were performed to analyze cellular responses and cytokine production after immunization by obtaining peripheral blood mononuclear cells (PBMC) and stimulating with *Pf* specific antigens, whole SPZ, or infected RBC. For *Pf* GAP2KO studies, interferon-γ (IFN-γ) producing CD8^+^ T cell responses increased after immunization (15). For PfSPZ Vaccine immunization, levels of CD8^+^ T cells that produced effector cytokine molecules [IFN-γ, interleuken-2 (IL-2), or transcription factor-alpha (TNF-α)] increased compared to pre-vaccination (22, 23). However, cytokine-producing T cell responses did not correlate with protection (22, 23).

Non-recirculating liver resident CD8^+^ T cells represent an immunological marker that cannot be sampled in peripherally circulating cell populations (e.g., PBMC) from human clinical trials. Several studies support liver-resident and IFN-γ-producing CD8^+^ T cells in providing protection after WSV administration. First, a PfSPZ immunization study was performed and involved different vaccine administration routes in mice, non-human primates (NHP), and humans (24). The greatest protection after CHMI was provided by IV vaccination compared to intradermal or intramuscular. Also, animal studies in mice and NHP identified IFN-γ-producing CD8^+^ T cells in the liver, which correlated with protection by IV administration (24). Subsequent parabiosis experiments in mice confirmed the existence of liver-resident CD8^+^ T cells (25), which provided RAS-induced malaria immunity (26). Further PfSPZ vaccination studies of NHP support liver resident IFN-γ-producing CD8^+^ T cells in providing immunity as these cell levels were ~100-fold higher in liver than in blood (27). Finally, several pre-clinical and clinical PfSPZ and CVac studies have also identified γδ T cells as possible correlates of protection that increase after subsequent vaccinations (22, 23, 27, 28).

While WSV have reduced immunogenicity and efficacy in some pre-exposed individuals (7, 29), vaccination strategies are underway to improve WSV immunogenicity. This work was motivated by numerous studies that support liver-resident CD8^+^ T cells as primary mediators of long-term pre-erythrocytic immunity. Several groups are developing either “prime and target” or “prime and trap” approaches in which peripheral CD8^+^ T cells are first primed and then targeting to the liver by high expression of *Plasmodium* subunit antigens or RAS infection (30, 31). These methods lead to higher levels of liver-resident CD8^+^ T cells and improved RAS or subunit vaccine efficacy compared to RAS or subunit alone (30, 31). Further clinical studies will determine whether these pre-clinical results translate to protect diverse populations of humans, especially in malarious regions.

Additional WSV immunogenicity studies must be performed to identify strong immune correlates for the purpose of guiding clinical trials, including dose and regimen strategies. These studies may be focused on identifying antigen specificity of important T cell populations (γδ and liver resident CD8^+^ T cells). For example, sequencing studies of T-cell receptors that bind malaria-specific antigens may identify strong correlates of protection. Moreover, since liver resident T-cells cannot be studied using PBMC from human clinical trials, future studies using NHP will prove valuable in identifying strong immune correlates of protection provided by WSV.

### *In vitro* Sporozoites for *Pf* Malaria Vaccines

While WSV are the most effective vaccines against *Pf* malaria, major hurdles prevent the manufacturing of sufficient quantities at a commercial scale. Current methods of vaccination with SPZ include delivery by mosquito bite or by injection after technically challenging and laborious mosquito dissections. Neither approach is practical for large-scale or cost-effective vaccination, which is required for commercialization. Culturing systems that produce infective SPZ independent of mosquitoes would enable scalable production of *Pf* SPZ for WSV.

### *Plasmodium* Stage Development in Mosquitoes

Continuous culture of blood stage malaria parasites (asexual and sexual) has been established for decades (32–35). However, the *in vitro* culturing of mosquito stages has proven extremely challenging. To culture SPZ, one must understand *Pf* development within the mosquito and recapitulate this in culture. *Pf* develops through five sequential stages in mosquitoes: gamete, zygote, ookinete, oocyst, and SPZ (Figure 1). Prior to mosquito stages, gametocytes are ingested by mosquitoes during a blood meal. After ingestion, gametocytes within the mosquito midgut emerge and transform into gametes that fuse to form a zygote. The zygote undergoes DNA replication and maturation into a motile and invasive ookinete that traverses the mosquito peritrophic membrane and midgut epithelium, and finally embeds into the basal lamina on the midgut periphery. The interaction between the ookinete and the mosquito midgut basal lamina is thought to trigger the transformation into the early oocyst (36–38). The sessile oocyst is the site of SPZ development (up to several thousand SPZ per mature oocyst). Once released, SPZ travel to the salivary glands through the hemocoel where they can be inoculated into a host during a mosquito blood meal (39).

As *Plasmodium* develops through multiple life cycle stages, studies indicate that transitions are driven by coordinated changes in stage-specific gene expression controlled by the apicomplexan Apetala2 (AP2) family of transcription factors (TFs) (40, 41). The 26 AP2 TFs represent the only known family of *Plasmodia* TFs and contain at least one AP2 DNA-binding domain (42). TFs controlling stage transitions include AP2-O, AP2-O2, AP2-O3, and AP2-O4 for ookinetes and oocysts (41, 43, 44), AP2-Sp, AP2-Sp2, and AP2-Sp3 for mature oocysts and/or SPZ (41, 45), AP2-G, and AP2-G2 for gametocytes (41, 46, 47), and AP2-L for liver stages (48). These TFs co-regulate expression of genes with common *cis* promoter elements enabling control of the *Plasmodium* life cycle (41). Understanding stage transition through gene regulation could improve SPZ culturing systems. Other culturing system improvements could include applying knowledge of the parasite's environment while developing in mosquitoes.

### *Plasmodium in vitro* Sporozoite Culturing Systems

Although several publications already exist that describe *in vitro* culturing of *Plasmodium* SPZ for the mouse (*P. berghei, P. yoelii*), avian (*P. gallinaceum*), and human (*Pf*) species (49–52), there is only a single publication outlining culturing methods for each species and SPZ characterization. Specific details on the different *Plasmodium* culturing systems are provided in Table 1. Culturing system factors that likely support production of SPZ include co-culturing with *Drosophila* support cells (L2/S2), an optimal medium (Roswell Park Memorial Institute [RPMI] or Schneider's), and culturing on a gel-like surrogate for the mosquito basal lamina. All culturing systems required *Drosophila* co-culturing or using conditioned media (spent media) from L2/S2 cells. *Drosophila* cells may provide nutrients for the developing oocyst and/or oocyst capsule, including soluble nutrients, growth/signaling factors and/or extracellular matrix molecules that are normally present in the mosquito midgut basal lamina or hemolymph. For culturing media, the avian and human culturing systems utilized RPMI, which is also used for *Pf* blood stage culture. Subsequent studies on culturing the murine-specific *Plasmodia* determined that Schneider's media optimally supported *Drosophila* cell growth (49). Finally, for the basal lamina, all culturing systems except the *P. yoelii* system found the necessity of Matrigel, which is a basal lamina-like gel derived from murine Engleberth-Holm-Swarm tumor cells and consists of laminin, collagen type IV, heparan sulfate proteoglycan, entactin, and growth factors. Laminin and collagen IV are known components of the mosquito basal lamina and are considered important for ookinete binding and oocyst transformation for mosquito and *in vitro* development (36–38, 56). In sum, there are shared characteristics of the *Plasmodium in vitro* sporozoite (IVS) culturing systems that reveal important factors supporting axenic SPZ development, including *Drosophila* cells, Schneider's media, and Matrigel or a similar basal lamina.

**Table 1:** Characteristics of *in vitro* sporozoite culturing systems for *Plasmodium gallinaceum, falciparum, berghei* and *yoelli*.

***Plasmodium* species (reference)**	**Oocyst /SPZ culture conditions**	**Basal lamina requirement**	**Oocyst diameter**	**Ookinete to oocyst conversion *in vitro* (early or mature)**	**SPZ days observed**	**SPZ characterization**
				***in vivo* provided for comparison**	
*P. gallinaceum*	*Drosophila* cells required	Matrigel	Day 3–7 mm for elongate	10–30% (mature)	Days 10 to 22 peaking on Day 16	Morphology with anti-CSP staining
(51)	In RPMI media		Days 5 to 7–up to 30 mm for spherical and up to 40 mm for elongated	1–21^*^ (53)		
*P. falciparum*	*Drosophila* cells required	Matrigel	Day 7–15 to 20 mm	N.D.	Days 12 to 16	Morphology with anti-CSP staining
(52)	In RPMI media		Days 10 to 12–25 to 40 mm	0.4–1.3%^*^ (54)		
*P. berghei*	*Drosophila* cells required	Matrigel	Day 15–up to 40 mm	68% (mature)	Days 15 to 28	Morphology with Giemsa staining; Infectivity in
(49)	In Schneider's media			0–18%^*^ (53)		hepatocytes of mice observed with subsequent blood stage transition and then mosquito infection
*P. yoelii*	*Drosophila* cells or conditioned media required	Matrigel not required	Day 3–4 mm	7.1% (early)	Days 6 to 30	Morphology with anti-CSP staining; Infectivity of primary mouse hepatocytes and
(50)	In Scheider's media		Days 6 to 7–10 mm	0–75%^*^ (55)		hepatocytes of mice observed with subsequent blood stage transition

*The features, including culture conditions, parasites observed, conversion frequency from ookinete to oocyst, and details of SPZ characterization are outlined and compared between culturing systems. For oocyst diameter, the observed oocysts were spherical unless otherwise noted. N.D, not described; RPMI, Roswell Park Memorial Institute*.

* *The range is due to differences in Anopheles mosquito species tested*.

### Characterization of Cultured Oocysts and Sporozoites

For the different *Plasmodium* culturing systems, oocyst, and SPZ development have been characterized to varying degrees (Table 1). The study that describes *Pf* culturing demonstrated that IVS morphology was similar to mosquito-derived SPZ and that IVS expressed the major SPZ surface protein, CSP (52). However, IVS functionality in terms of motility or hepatocyte traversal/invasion/infection was not analyzed. Similarly, for *P. gallinaceum*, only morphology and CSP expression were described (51). Separate studies on the rodent malaria parasites, *P. berghei* (49), and *P. yoelii* (50) analyzed morphology and sporozoite invasion of hepatocytes in mice. For *P. berghei*, IVS were produced with similar morphology and mouse infectivity compared to mosquito-derived SPZ, and blood stage infection was also observed (49). For *P. yoelii*, IVS infected primary hepatocytes in culture at a low rate (1/10,000) and hepatocytes in mice but infectivity was reduced compared to mosquito-derived SPZ. Furthermore, blood stage parasites were only seen in 1/15 infected mice (50).

While these studies demonstrate the feasibility of producing IVS that are capable of hepatocyte infection, many researchers have failed to replicate mosquito-stage culturing of *Pf* and other *Plasmodium* species due to the complexity of *Plasmodium* mosquito stage development and the extreme scientific and technical challenges of establishing culturing systems. We and others, including Sanaria, have made further improvements on developing *Pf* IVS culturing systems to ultimately produce SPZ for use in WSV development. For example, our *Pf* culturing system uses the unique nutrient, insect (silk-worm) hemolymph (56). Oocysts and maturing SPZ may receive soluble nutrients, growth factors, and/or signaling factors from the insect hemolymph as from the mosquito hemolymph. Successful *in vitro Pf* conversion frequencies compared to mosquito development have been achieved for life cycle stages up to the mature oocyst stage (56). Current developing approaches are focused on improving conversion to the mature oocyst and SPZ stages by using 3-dimensional culturing on non-Matrigel basal lamina-like substrates. Matrigel presents challenges for cGMP vaccine production as it is tumor-derived and may vary between batches.

### Summary and Future Considerations

Future hurdles for the production of IVS to use in WSV include demonstrating equivalent functionality compared to mosquito-derived SPZ in terms of hepatocyte invasion/infection using cell lines and human-liver chimeric mouse models (57). While *P. berghei* IVS exhibit infectivity comparable to mosquito-derived SPZ, *P. yoelii* IVS have decreased infectivity, and hepatocyte infectivity was not assessed for *Pf* or *P. gallinaceum* IVS. IVS may resemble immature mosquito midgut SPZ more so than fully infectious mosquito salivary gland SPZ, which further develop in the mosquito hemocoel and salivary gland (58). Additional cues may be necessary for the IVS culturing systems to produce fully infectious, mature SPZ for use as a WSV. Moreover, GAP or RAS IVS will also need to satisfy safety requirements in terms of liver stage arrest and demonstrate absence of toxins, pathogens, or other contaminants. Thus, hurdles must be overcome in the purification of IVS from the 3D basal lamina and from *Drosophila* cells while maintaining IVS functionality. If these issues can be solved, culturing of SPZ would provide scalable production of WSV for further development as *Pf* malaria vaccines.

## Author Contributions

LSI, YZ, JD, AMG, AMV, and AKG developed the manuscript. LSI and AKG drafted the manuscript. All authors read and approved the final manuscript.

### Conflict of Interest Statement

YZ, JD, AMG, and AKG are employed by a malaria vaccine company, MalarVx, Inc. LSI was employed at MalarVx, Inc. at the time of the study. The remaining author declares that the research was conducted in the absence of any commercial or financial relationships that could be construed as a potential conflict of interest.

## Abbreviations

CHMI: controlled human malaria infection
CPS: chemoprophylaxis with *Pf* sporozoites
CSP: circumsporozoite protein
CVac: CPS using Sanaria's injectable sporozoites
GAP: genetically attenuated parasite
IFN-γ: interferon-γ
IL-2: interleuken-2
ITV: infection treatment vaccination
IVS: *in vitro* sporozoite
NHP: non-human primate
Pf: *Plasmodium falciparum*
PMBC: peripheral blood mononuclear cells
RAS: radiation attenuated sporozoite
RBC: red blood cell
SPZ: sporozoite
RPMI: Roswell Park Memorial Institute
RTS, S, RTS: S/AS-01
SPZ: sporozoite
TNF-α: transcription factor α
WHO: World Health Organization
WSV: whole sporozoite vaccine.
